# Chemically modified h*CFTR* mRNAs recuperate lung function in a mouse model of cystic fibrosis

**DOI:** 10.1038/s41598-018-34960-0

**Published:** 2018-11-13

**Authors:** A. K. M. Ashiqul Haque, Alexander Dewerth, Justin S. Antony, Joachim Riethmüller, Georg R. Schweizer, Petra Weinmann, Ngadhnjim Latifi, Hanzey Yasar, Nicoletta Pedemonte, Elvira Sondo, Brian Weidensee, Anjali Ralhan, Julie Laval, Patrick Schlegel, Christian Seitz, Brigitta Loretz, Claus-Michael Lehr, Rupert Handgretinger, Michael S. D. Kormann

**Affiliations:** 1 Department of Pediatrics I – Pediatric Infectiology and Immunology, Translational Genomics and Gene Therapy, University of Tuebingen, Tuebingen, Germany; 2 Department of Pediatrics I - Cystic Fibrosis Ambulance, Tuebingen, Germany; 3 Helmholtz Institute for Pharmaceutical Research Saarland (HIPS), Helmholtz Center for Infection Research (HZI), Saarbruecken, Germany; 4 U.O.C. Genetica Medica, Istituto Giannina Gaslini, Genova, Italy; 5 Department of Pediatrics I - Immunology and Pneumology/Cystic fibrosis, Department of Pediatrics I, University of Tuebingen, Tuebingen, Germany; 6 Department of Pharmacy, Saarland University, Saarbruecken, Germany; 7 Department of Hematology, Oncology, Clinical Immunology, University of Tuebingen, Tuebingen, Germany

## Abstract

Gene therapy has always been a promising therapeutic approach for Cystic Fibrosis (CF). However, numerous trials using DNA or viral vectors encoding the correct protein resulted in a general low efficacy. In the last years, chemically modified messenger RNA (cmRNA) has been proven to be a highly potent, pulmonary drug. Consequently, we first explored the expression, function and immunogenicity of human (h)CFTR encoded by cmRNA^h*CFTR*^ *in vitro* and *ex vivo*, quantified the expression by flow cytometry, determined its function using a YFP based assay and checked the immune response in human whole blood. Similarly, we examined the function of cmRNA^h*CFTR*^ *in vivo* after intratracheal (i.t.) or intravenous (i.v.) injection of the assembled cmRNA^h*CFTR*^ together with Chitosan-coated PLGA (poly-D, L-lactide-co-glycolide 75:25 (Resomer RG 752 H)) nanoparticles (NPs) by FlexiVent. The amount of expression of human hCFTR encoded by cmRNA^h*CFTR*^ was quantified by hCFTR ELISA, and cmRNA^h*CFTR*^ values were assessed by RT-qPCR. Thereby, we observed a significant improvement of lung function, especially in regards to FEV_0.1_, suggesting NP-cmRNA^h*CFTR*^ as promising therapeutic option for CF patients independent of their *CFTR* genotype.

## Introduction

Cystic fibrosis (CF), the most common life-limiting autosomal-recessive disease in the Caucasian population (1/2,500 newborns), affects more than 80,000 people worldwide^1^. It is caused by different mutations within the gene encoding for the CF transmembrane conductance regulator (CFTR). Those mutations result in impaired anion secretion and hyper-absorption of sodium ions across epithelia^2,3^. Chronic lung disease and slow lung degradation are the major contributing factors to both mortality and strongly reduced quality of life^4,5^. With currently available therapies, the mean survival is between 35 and 45 years^6,7^. Since the CFTR gene was first cloned in 1989, many efforts have been made to deal with the mutations at a cellular and genetic level^8,9^. Gene therapy approaches made it quickly to the clinic aiming to deliver viral CFTR-encoding vectors (such as adenoviruses (Ad) or adeno-associated viruses (AAV)) to CF patients^10^. However, none of the clinical studies and current treatments seem to provide sufficient human (h)CFTR expression to prevent the ultimately lethal CF symptoms in the respiratory tract of CF patients. Furthermore, repeated administration of viral vectors or DNA may lead to the development of unwanted immune reactions, mainly due to viral capsids and vector-encoded proteins^10–12^.

Newly designed viral vectors circumvent those problems and can be administered repeatedly, but from a clinical perspective, the field is still in need of a therapeutic tool that combines efficient expression in lungs and other (affected) organs and cells while avoiding immunogenicity and genotoxicity completely^13–15^. The non-viral *CFTR*-encoding plasmid–liposome complex pGM169/GL67A has been one of the most promising therapeutical approach used in clinical trial by Alton’s group. Beside some encouraging results, the trial only managed to modestly improve forced expiratory volume in 1 s (FEV_1_) after repeated administration along with no improvement in patient’s quality of life^16,17^. Recently, *in vitro* transcribed (IVT) chemically modified messenger RNA (cmRNA) came into focus, which has the potential to combine striking advantages in a single-stranded molecule^18,19^. Chemically modified mRNA has been tested for repeated administration, without developing immune responses or losing efficacy, presenting cmRNA^h*CFTR*^ complexed with biodegradable chitosan-coated PLGA nanoparticles (NPs) as a promising therapeutic for the treatment of CF patients^19–21^. Versatile delivery options of mRNA ensure the unique possibility to utilize NP-cmRNA^h*CFTR*^ in early infants as well as adults, independent of the underlying *CFTR* mutation. To the best of our knowledge, we provide the first *in vivo* studies delivering cmRNA^h*CFTR*^ to the lungs of CFTR deficient mice (*Cftr*^−/−^) by intravenous (i.v.) and intratracheal (i.t.) administration, complexed with NPs. We provide a proof of concept of NP- cmRNA^h*CFTR*^ mediated, ELISA quantified, hCFTR expression in the lungs of *Cftr*^−/−^ mice, leading to significantly reduced chloride secretion and, more importantly, restored criticial lung function parameters, including the most important parameter to evaluate mortality and morbidity of CF patients, the forced expiratory volume (FEV) in 1s or 0.1s in small animals, respectively^22–24^.

## Materials and Methods

### mRNA production

h*CFTR* was PCR amplified from pcDNA3.hCFTR with primers adding *Nhe*I (Fwd: 5′-TTAGCTAGATGCAGAGGTCGCCTC-3′) and *Kpn*I (Rev: 5′-GCGGGTACCTATCTTGCATCTCTTCT -3′) restriction sites to each end. The PCR product was cloned into a poly(A)-120 containing pVAX (pVAX.A120, www.lifetechnologies.com) by sticky-end ligation using the mentioned restriction sites. pVAX.A120 containing h*CFTR* is referred as pDNA^h*CFTR*^ throughout this study. For control experiments, DsRed reporter protein was sub-cloned into pVAX.A120 vector from its original vector pDsRed (www.clontech.com). For *in vitro* transcription (IVT), the plasmids were linearized downstream of the poly(A) tail with *Xho*I (www.neb.com). IVT reaction was carried out using MEGAscript T7 Transcription kit (www.ambion.com) with an anti-reverse CAP analog (ARCA) at the 5′ end (www.trilink.com). To produce chemically modified mRNA, the following chemically modified nucleosides were added to the IVT reaction in the indicated ratios: uridine-tri-phosphate (UTP) and cytidine-tri-phosphate (CTP) were fully replaced by N1-Methylpseudo-UTP and 5-Methyl-CTP, abbreviated to ${{\rm{cmRNA}}}_{{\rm{N}}1{{\rm{\Psi }}}_{1.0}/{{\rm{m5C}}}_{1.0}}^{{\rm{h}}{CFTR}}$ and partly replaced by the incorporation of 25% 2-Thio-UTP and 25% 5-Methyl-CTP, respectively, abbreviated to ${{\rm{c}}{\rm{m}}{\rm{R}}{\rm{N}}{\rm{A}}}_{{\rm{s}}2{{\rm{U}}}_{0.25}{/{\rm{m}}5{\rm{C}}}_{0.25}}^{{\rm{h}}CFTR}$ (www.trilink.com). The cmRNA^h*CFTR*^ and cmRNA^*DsRed*^ were purified using the MEGAclear kit (www.ambion.com) and analyzed for size and concentration using an RNA NanoChip 6000 for Agilent 2100 Bioanalyzer (Supplement, Fig. S1) (ww.agilent.com).

### Cell culture and Transfection

CFBE41o− and 16HBE14o- cells (from Gruenert’s lab) were incubated at 37 °C in a humidified atmosphere containing 5% CO_2_ until they reached 80–90% confluency. Cell lines were washed with cold, sterile PBS and detached by Trypsin-EDTA. Trypsinization was stopped by adding minimum essential medium (MEM; www.thermofisher.com) containing 10% fetal calf serum. Cells were collected and spun down at 500 × g for 5 minutes before resuspension in fresh MEM. One day before transfection, 250,000 cells/well/1 ml were plated in 12-well plates and grown overnight in MEM without antibiotics. At confluence of 70–90%, cells were then transfected with 1000 ng (c)mRNA^h*CFTR*^ or equivalent (in nmol) pDNA^h*CFTR*^ using Lipofectamine 2000 (www.invitrogen.com) following the manufacturer’s instructions and after changing the media to the reduced serum media, Opti-MEM (www.thermofisher.com). After 5 hours, the complexes were removed by replacement with fresh culture medium. Cells were kept for 24 h and 72 h before further analyses.

### Flow cytometry analyses

All flow cytometry analyses were performed using a BD LSR Fortessa X-20 SORP (www.bdbioscience.com). For detection of hCFTR protein in 16HBE14o- and CFBE41o− cell lines, cells were transfected as described above and subsequently prepared for intracellular staining using a Fixation/Permeabilization Solution Kit as directed in the manufacturer’s instruction (www.bdbioscience.com). As primary antibody mouse anti-human hCFTR clone 596 (1:500, kindly provided by the cystic fibrosis foundation therapeutics Inc.) has been used. As secondary antibody served Alexa Fluor 488 goat anti-mouse IgG (1:1,000, www.lifetechnologies.com). At least 20,000 gated cells per tube were counted. Data were analyzed with FlowJo software, version 10.

### Western blot analysis

Protein isolated from cell lines was separated on Bolt NuPAGE 4–12% Bis-Tris Plus gels and a Bolt Mini Gel Tank (all from www.lifetechnologies.com). Immunoblotting for hCFTR was performed by standard procedures according to the manufacturer’s instructions using the XCell II Mini-Cell and blot modules (www.lifetechnologies.com). After blocking for 1 h in 5% dry milk at room temperature, primary antibody against hCFTR clone 596 (1:500, kindly provided by the cystic fibrosis foundation therapeutics Inc.) or anti-GAPDH (1:1000) (www.scbt.com) was incubated overnight, horseradish peroxidase–conjugated secondary antibodies (anti-mouse from www.dianova.com) were incubated for 1 h at room temperature. All blots were processed by using ECL Prime Western Blot Detection Reagents for 30 min exposure time (www.gelifesciences.com). Semiquantitative analysis was performed using the ImageJ software and overexposure has been avoided as per as digital image and integrity policies.

### Immunofluorescence

CFBE41o− and 16HBE14o- were plated on a cell culture insert (0.75 × 10^6^ cells per insert) containing a PET membrane (0.4 μm pore size) (www.corning.com) to provide an air-liquid interface. Cells were transfected 12 h after plating with 5000 ng cmRNA^h*CFTR*^ or equivalent (in nmol) pDNA^h*CFTR*^ using Lipofectamine 2000 (www.invitrogen.com) according to manufacturer’s instructions. Membranes were cut out from the insert 24 h after transfection, fixed with 4% PFA, blocked with 0.1% BSA and Fc blocker. Blocking was followed by overnight incubation with hCFTR clone 596 (1:250, kindly provided by the cystic fibrosis foundation therapeutics Inc.). As secondary antibody served Alexa Fluor 594 goat anti-mouse IgG (1:250, www.abcam.com, (ab150116)). Membranes were mounted on a coverslip and images were acquired by Zeiss Confocal Laser Scanning Microscope (CLSM) 710 NLO with Zen software.

### YFP-based functional assay

CFTR activity following transient transfection of (c)mRNA^h*CFTR*^ in A549 and CFBE41o− cells was determined using the halide-sensitive yellow fluorescent protein YFP-H148Q/I152L^25^. CFTR deficient A549 or CFBE14o- cells stably expressing the YFP were plated in 96-well microplates (50,000 cells/well) in 100 µl of antibiotic-free culture medium and, after 6 h, transfected (in case of CFBE14o- reverse transfected) with either plasmids carrying the coding sequence for CFTR or different (c)mRNA^h*CFTR*^. For each well, 0.25 µg of mRNA or plasmid DNA and 0.25 µl of Lipofectamine 2000 were pre-mixed in 10 µl of Opti-MEM (www.invitrogen.com) to generate transfection complexes that were then added to the cells. After 24 hours, the complexes were removed by replacement with fresh culture medium. The CFTR functional assay was carried out 24, 48 or 72 h after transfection. For this purpose, the cells were washed with PBS and incubated for 20–30 min with 60 µl PBS containing forskolin (20 µM). After incubation, cells were transferred to a microplate reader (FluoStar Galaxy; www.bmg.labtech.com) for CFTR activity determination. The plate reader was equipped with high-quality excitation (HQ500/20X: 500 ± 10 nm) and emission (HQ535/30M: 535 ± 15 nm) filters for yellow fluorescent protein (www.chroma.com). Each assay consisted of a continuous 14-s fluorescence reading (5 points per second) with 2 s before and 12 s after injection of 165 µl of a modified PBS containing 137 mM NaI instead of NaCl, resulting in a final 100 mM NaI concentration in the well). To determine iodide (I^−^) influx rate, the final 11 s of the data for each well were fitted with an exponential function to extrapolate initial slope. After background subtraction, cell fluorescence recordings were normalized for the initial average value measured before addition of I^−^. Maximum slopes were converted to rates of variation of intracellular I^−^ concentration (in mM/s) using the equation: d[I^−^]/dt = K_I_[d(F/F_0_)]/dt, where K_I_ is the affinity constant of YFP for I^−^, and F/F_0_ is the ratio of the cell fluorescence at a given time vs. initial fluorescence^25^.

### Whole blood assay

Ethical approval for using whole blood from healthy donor was obtained from Ethics Commission University Clinic of Tuebingen, Germany (349/2013BO2) and experiments were conducted in accordance with relevant guidelines and regulations. Informed consent form (following WHO guideline) was signed by each volunteer (healthy donor) and kept safely by principal investigator for privacy requirement. Blood samples from three healthy donors were collected in EDTA collection tubes (www.sarstedt.com). For each treatment group, 2 ml of EDTA-blood was transferred into 12-well plates and treated accordingly. R848 (Resiquimod, www.sigmaaldrich.com) was added at a concentration of 1 mg/ml to the respective blood positive controls. cmRNA^h*CFTR*^ and pDNA^h*CFTR*^ (7 picomol each) were complexed to NPs at a ratio of 1:10. The samples were kept at 37 °C incubator maintaining 5% CO_2_. At 6 h and 24 h, 1 ml of whole blood was transferred into microtubes containing serum gel (www.sarstedt.com) and spun down at 10,000 × g for 5 min to obtain serum. Sera were stored at −20 °C for further cytokine measurement analyses. Serum was used to conduct IFN-α, TNF-α and IL-8 ELISA at manufacturer’s instruction (www.thermofisher.com).

### Animal experiments

All animal experiments were approved by Regierungspräsidium Tübingen, Baden-Württemberg and carried out according to the guidelines of the German Law for the Protection of Animals (File Number: 35/9185.81-2/K3/16). *Cftr*^−/−^ mice (CFTR^tm1Unc^) were purchased from Jackson Laboratory (www.jax.org) at the age of 6 to 8 weeks and were maintained under standardized specific pathogen-free conditions on a 12 h light-dark cycle. Food, water as well as nesting material were provided *ad libitum*. Prior to i.t. spray applications, mice were anesthetized intraperitoneally (i.p.) with a mixture of medetomidine (0.5 mg/kg), midazolam (5 mg/kg) and fentanyl (50 μg/kg). *Cftr*^−/−^ mice received 40 μg or 80 µg of cmRNA^h*CFTR*^ or an equivalent amount (calculated using nmols) pDNA^h*CFTR*^ complexed with chitosan-coated PLGA nanoparticles [Chitosan (83% deacetylated (Protasan UP CL 113) coated PLGA (poly-D,L-lactide-co-glycolide 75:25 (Resomer RG 752 H)) nanoparticles; short: NPs] (Full details of nanoparticles are provided in Supplement Table T1 and Fig. S5) by intratracheal (i.t.) spraying (n = 4), and intravenous (i.v.) injection (n = 4–7) into the tail vein. Mock-treated control *Cftr*^−/−^ mice received 40 μg or 80 μg cmRNA^*DsRed*^ complexed to NPs (n = 5) by i.v. or i.t administration, respectively, or just 200 μl NPs by both i.v. and i.t. delivery. An antidote with a mixture of naloxone (1.2 mg/kg), flumazenil (0.5 mg/kg) and atipamezol (2.5 mg/kg) was used against anesthetizing reagents. For both interventions, NP-cmRNA and NP-pDNA complexes were administered in a total volume of 200 μl, twice at an interval of 3 days (day 0 and day 3). After 6 days, mice were sacrificed for endpoint analyses. A detailed description of the i.t. procedures are explained in previously published study^26^.

### Pulmonary mechanics

Lung function for each group was evaluated using a FlexiVent^®^ equipped with FX1 module and NPFE extension and was operated by the flexiWare v7.2 software (www.scireq.com). Prior to tracheostomy, mice were anesthetized intraperitoneally as described above. After anesthesia, a 0.5 cm incision was performed in rostral to caudal direction. A flap of skin was retracted, the connective tissue was dissected, and the trachea was exposed. The trachea was then cannulated between the second and third cartilage ring with a blunt-end stub adapter. The mouse was connected to the FlexiVent^®^ system and quasi-sinusoidally ventilated^27^ with a tidal volume of 10 ml/kg. A breathing frequency of 150 breaths per min was maintained with an inspiratory to expiratory ratio of 2:3.

Airway resistance (Rn), which is dominated by the resistance of the large conducting airways was considered in this study when the coefficient of determination of the model fit was ≥0.9. Compliance (Cst) was calculated straight from deflating arm of the pressure volume (PV) loops and ramp style pressure-driven maneuver (PVr-P). For obtaining FEV_0.1_ data a NPFE maneuver was performed which results in FV loops and FE-related parameters. The mice lung was inflated by a pressure of +30 cmH_2_O over 1.2 s and rapidly deflated to a negative pressure of −55 cmH_2_O to generate an imposed negative expiratory pressure gradient.

### Salivary assay

Prior to tracheostomy, anesthetized mice were injected with 50 µl of 1 mM acetylcholine (ACh) in the cheek to stimulate the production of saliva. The fluid was collected via glass capillaries and a chloride assay was performed using the Chloride (Cl^−^) Assay Kit according to the manufacturer’s protocol (www.sigmaaldrich.com). Briefly, saliva was diluted at a ratio of 1:100 with water in a total volume of 50 µl and subsequently 150 µl chloride reagent was added. After 15 min incubation at room temperature in the dark, absorbance was measured at 620 nm using an Ensight Multimode plate reader (www.perkinelmer.com).

### Enzyme-linked immunosorbent assays (ELISAs)

To detect protein levels of hCFTR after i.t. or i.v. injection of differently modified cmRNA^h*CFTR*^ in *Cftr*^−/−^ mice (CFTR^tm1Unc^), the lungs were isolated at day 6 (experimental endpoint), homogenized and lysed in 600 µl RIPA-buffer and 5 µl protease inhibitor cocktail with tubes of the Precellys Ceramic Kit 1.4/2.8 mm at 6,500 rpm for 10 s for a total of three cycles, each interrupted by a 15 s break in a Precellys Evolution Homogenizer for protein isolation (all from www.peqlab.com). Subsequently, supernatants were kept on ice and additionally homogenized 10 times with a 20G needle and incubated for 20 min (www.bdbioscience.com). Lysates were spun down for 20 min at 13,000 × g and 4 °C. The supernatant was collected and stored at −20 °C for further use. Prior to hCFTR ELISA detection, protein concentration was measured using the Pierce BCA protein assay kit (www.thermofisher.com). For each sample, an equal amount of 15 µg whole protein lysate was used. A human CFTR ELISA kit (www.elabscience.com) was used for hCFTR detection according to manufacturer’s instructions.

### Real-time RT-PCR

After i.t. or i.v. injection of cmRNA^h*CFTR*^ the lungs were isolated at day 6 (experimental endpoint), homogenized and lysed with tubes of the Precellys Ceramic Kit 1.4/2.8 mm at 5,000 rpm for 20 s in a Precellys Evolution Homogenizer for subsequent RNA-isolation (all from www.peqlab.com). Reverse transcription of 200 ng RNA was carried out using an iScript cDNA synthesis kit (www.bio-rad.com) and 1:20 dilution of the cDNA product had been used for further experiment. Detection of mRNA^h*CFTR*^ was performed by SYBR-Green based quantitative Real-time PCR in 15 μl reactions on a ViiA7 (www.lifetechnologies.com). In all involved procedures, we strictly followed the MIQE protocols for RealTime experiments^28^. Pre- and post-reaction rooms were strictly separated. Reactions were incubated for 10 min at 95 °C, followed by 40 cycles of 15 s at 95 °C and 2 min at 50 °C (annealing and extension), followed by standard melting curve analysis. The following primer pairs were used:

hCFTR fwd 5′-GAGATGCTCCTGTCTCCTGG-3′, rev 5′-CCTCTCCCTGCTCAGAATCT-3′; 18S rRNA fwd 5′-GGGAGCCTGAGAAACGGC-3′, rev 5′-GACTTGCCCTCCAATGGATCC-3′. Differences in mRNA expression between groups were analyzed by pair-wise fixed reallocation randomization tests with REST 2009 software after collection of the data from Viia7.

### Immune response *in vivo*

To assess immune responses to (c)mRNA^h*CFTR*^ and pDNA^h*CFTR*^, C57BL/6 (Jackson Laboratory (www.jax.org)) mice (n = 4 per group) were treated as described for *Cftr*^−/−^ mice. As positive controls a group of mice received two administrations of *E*. *coli* mRNA-NPs (20 µg) i.v. or i.t. C57BL/6 mice received two injections of 20 µg cmRNA^h*CFTR*^ complexed to NPs i.v. or i.t. After 6 h, 24 h, and 72 h of second injection mice were sacrificed and blood was collected. For cytokine measurement, blood from mice was used to obtain serum using a serum separator (www.sarstedt.com) and tested for IFN-α and TNF-α production as directed in the manufacturer’s instructions (www.thermofisher.com).

### Statistics

All analyses were performed using the Kruskal-Wallis test with GraphPad Prism Version 6 (www.graphpad.com). Most of the data are represented as mean ± SD; box plot data are represented as a mean ± minimum to maximum values. P ≤ 0.05 was considered statistically significant.

## Results

### (c)mRNA^h*CFTR*^ and hCFTR protein quantification *in vitro*

**Figure 1:**
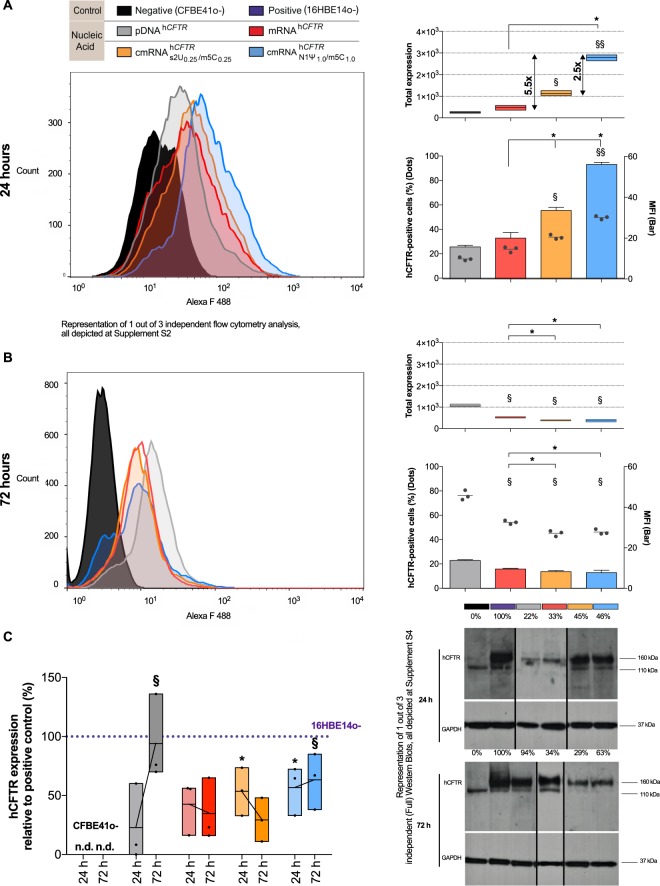
(c)mRNA^h*CFTR*^ and pDNA^h*CFTR*^ mediated expression of h*CFTR in vitr*o (**A**) Total expression of hCFTR (calculated by multiplying positive cells (dots) and MFI (bars)) 24 h after transfection with 1 µg (c)mRNA^h*CFTR*^ and equivalent nmols of pDNA^h*CFTR*^ detected by flow cytometry. (**B**) Total expression of hCFTR 72 h after transfection with 1 µg (c)mRNA^h*CFTR*^ and equivalent nmols of pDNA^h*CFTR*^ detected by flow cytometry. (**C**) Western Blots, semi-quantifying human CFTR in transfected CFBE41o- cells, normalized to GAPDH and put relative to CFTR levels in 16HBE14o- cells. Blot section cropped from different blots are delineated with clear dividing lines (black) and full blot of same exposure time (30 mins) are depicted in Supplement Fig. S4. All bar graph data are depicted as means ± SDs while box plots data are depicted as the means ± minimum to maximum values. *P ≤ 0.05 versus unmodified mRNA^h*CFTR*^; ^§^*P* ≤ 0.05 and ^§§^*P* ≤ 0.01 vs. pDNA^h*CFTR*^.

To evaluate the influence of chemical nucleoside modification, we first conducted a set of *in vitro* analyses to characterize the expression and functionality of hCFTR protein. First, we compared the expression profile of plasmid-encoded hCFTR (pDNA^h*CFTR*^), unmodified h*CFTR* mRNA (mRNA^h*CFTR*^) and two well-defined nucleoside modifications (${{\rm{cmRNA}}}_{{\rm{s}}2{{\rm{U}}}_{0.25}/{{\rm{m5C}}}_{0.25}}^{{\rm{h}}{CFTR}}$ and ${{\rm{cmRNA}}}_{{\rm{N}}1{{\rm{\Psi }}}_{1.0}/{\rm{m}}5{{\rm{C}}}_{1.0}}^{{\rm{h}}{CFTR}}$) which have been described to exert state-of-the-art stability/expression *in vitro* or lung-specific cell contexts *in vivo*^21,29–31^. Flow cytometry analyses 24 h after transfection of human cystic fibrosis bronchial epithelial (CFBE41o−) cells with pDNA^h*CFTR*^, mRNA^h*CFTR*^, ${{\rm{cmRNA}}}_{{\rm{s}}2{{\rm{U}}}_{0.25}/{{\rm{m5C}}}_{0.25}}^{{\rm{h}}{CFTR}}$ and ${{\rm{cmRNA}}}_{{\rm{N}}1{{\rm{\Psi }}}_{1.0}/{\rm{m}}5{{\rm{C}}}_{1.0}}^{{\rm{h}}{CFTR}}$, showed hCFTR positive cells (marked as black dots) ranging from 15.8% (pDNA^h*CFTR*^) to 49.6% (${{\rm{cmRNA}}}_{{\rm{N}}1{{\rm{\Psi }}}_{1.0}/{\rm{m}}5{{\rm{C}}}_{1.0}}^{{\rm{h}}{CFTR}}$) (*P* ≤ 0.01; Fig. 1A, lower panel). At 24 h, hCFTR positive cells and hCFTR median fluorescence intensities (MFIs, marked as columns) of (c)mRNA^h*CFTR*^ were significantly higher compared to pDNA^h*CFTR*^ (*P* ≤ 0.05; Fig. 1A, lower panel). At 24 h the total hCFTR expression, defined as median fluorescent intensity (MFI) multiplied by the transfection efficiency, of ${{\rm{cmRNA}}}_{{\rm{N}}1{{\rm{\Psi }}}_{1.0}/{\rm{m}}5{{\rm{C}}}_{1.0}}^{{\rm{h}}{CFTR}}$ was significantly higher compared to pDNA^h*CFTR*^ and mRNA^h*CFTR*^ (*P* ≤ 0.01; Fig. 1A, upper panel). In contrast, after 72 h (c)mRNA^h*CFTR*^ expressed significantly lower compared to pDNA^h*CFTR*^ transfected cells, reflected in the percentage of positive cells, MFI and in total hCFTR expression (*P* ≤ 0.05; Fig. 1B).

To confirm and substantiate those findings, we performed Western blot analyses of protein lysates taken from transfected CFBE41o- cells at 24 h and 72 h post treatment (Fig. 1C). As a positive control served protein lysate from untransfected 16HBE14o- cells, and GAPDH was used to normalize band intensities. At 24 h pDNA^h*CFTR*^ transfected CFBE41o- cells showed an average of 22.8% of the protein expression compared to hCFTR observed in 16HBE14o- cells, which increased 4.1-fold to 94.0% at 72 h (*P* ≤ 0.05; Fig. 1C). This drastic increase of hCFTR expression after pDNA transfection goes well in line with the observations in flow cytometry. As well as the quick onset of hCFTR expression after (c)mRNA^h*CFTR*^ transfection at 24 h (*P* ≤ 0.05; Fig. 1C). However, relative to the 24 h time-point, hCFTR expression after 72 h either remained nearly static (mRNA^h*CFTR*^ resulted in 33.8% and 34.7% expression at 24 h and 72 h, respectively), decreased (${{\rm{cmRNA}}}_{{\rm{s}}2{{\rm{U}}}_{0.25}/{{\rm{m5C}}}_{0.25}}^{{\rm{h}}{CFTR}}$ resulted in 45% and dropped to 29.3% hCFTR expression at 24 h and 72 h, respectively) or increased (${{\rm{cmRNA}}}_{{\rm{N}}1{{\rm{\Psi }}}_{1.0}/{\rm{m}}5{{\rm{C}}}_{1.0}}^{{\rm{h}}{CFTR}}$, 46.4% at 24 h and raised to 63.3% at 72 h). Ultimately, the expression of h*CFTR* mRNA *in vitro* was strongly dependent on its chemical modification, with ${{\rm{cmRNA}}}_{{\rm{N}}1{{\rm{\Psi }}}_{1.0}/{\rm{m}}5{{\rm{C}}}_{1.0}}^{{\rm{h}}{CFTR}}$ resulting in the most robust hCFTR expression among all (c)mRNA transfections (All the blots are separately provided in Supplement Fig. S4).

**Figure 2:**
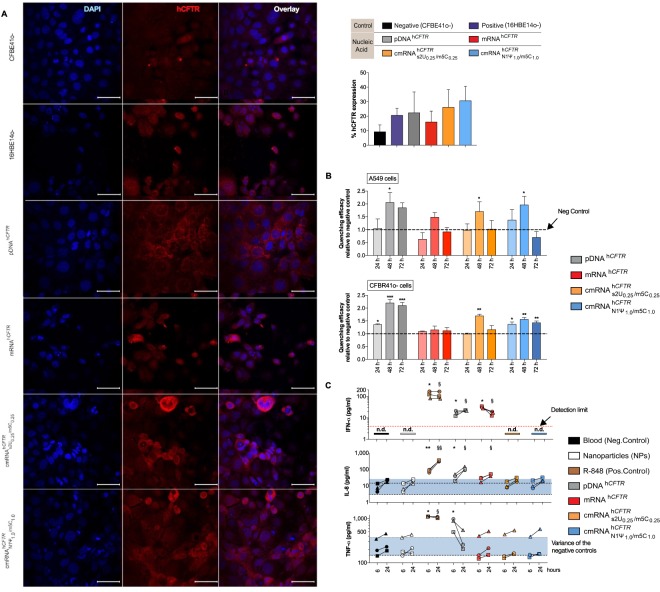
(c)mRNA^h*CFTR*^ and pDNA^h*CFTR*^ mediated expression of hCFTR by immunofluorescence and functional h*CFTR in vitro* and immunogenicity in human whole blood. (**A**) Detection of hCFTR protein by immunofluorescence (after 24 h), percent of hCFTR expression in pDNA^h*CFTR*^ or (c)mRNA^h*CFTR*^ transfected CFBE41o- cells compare to untransfected CFBE41o- and 16HBE14o- cells. Image J has been used for calculating means ± SDs of hCFTR positive cells; (**B**) Quenching efficacy of pDNA^h*CFTR*^ or (c)mRNA^h*CFTR*^ transfected CFBE41o- and CFTR null A549 cells relative to un-transfected controls was measured at 24 h, 48 h and 72 h post-transfection. *P ≤ 0.05 versus un-transfected controls; (**C**) 2 ml whole blood, each from three different healthy human donors, were incubated with either R848 (1 mg/ml) or 7 pmol pDNA^h*CFTR*^ or 7 pmol (c)mRNA^h*CFTR*^ (providing the same total number of nucleic acid molecules) and NPs at a 1:10 ratio; after 6 h and 24 h the immune response was determined by ELISA in the sera; The blue area represents the variance of the negative controls which are biological replicates. n.d., not detectable and red dotted lines mark the detection limit as specified in the respective ELISA kit. All bar graph data are depicted as means ± SDs while box plots data are depicted as the means ± minimum to maximum values. *and ^§^*P* ≤ 0.05 (^§§^*P* ≤ 0.01) versus control at 6 h and 24 h, respectively.

All *in vitro* results are also underlined by the conducted immunofluorescence imaging. All tested samples show a higher amount of hCFTR positive cells compared to the negative control (CFBE41o- cells; Fig. 2A). Additionally, transfection with unmodified mRNA^h*CFTR*^ produced a lower amount of hCFTR positive cells compared to both pDNA^h*CFTR*^ and cmRNA^h*CFTR*^ with the highest amount of hCFTR positive cells in the samples transfected with ${{\rm{cmRNA}}}_{{\rm{N}}1{{\rm{\Psi }}}_{1.0}/{\rm{m}}5{{\rm{C}}}_{1.0}}^{{\rm{h}}{CFTR}}$ (Fig. 2A). Looking at the fluorescence image itself transfection of pDNA^h*CFTR*^ shows a quite dispersed appearance of hCFTR within the cells compared to cmRNA^h*CFTR*^ transfection seeming to have a higher abundance of hCFTR towards the cell membrane (Fig. 2A, left panel). In general, the Immunofluorescence imaging confirms that transfection with pDNA^h*CFTR*^ as well as (c)mRNA^h*CFTR*^ leads to increased levels of hCFTR protein within the transfected cells.

### hCFTR (c)mRNA functionality test *in vitro*

For functional analysis of the (c)mRNA^h*CFTR*^ -encoded CFTR channel, we performed a YFP-based functional assay using CFTR null A549 cells or ΔF508 CFBE41o- cells which stably express halide-sensitive YFP-H148Q/I152L^30^. Quenching of the YFP signal induced by hCFTR channel-mediated I^−^ influx is reciprocally proportional to hCFTR channel function^25,32^. Figure 2B shows the quenching efficacy after transfection of 250 ng (c)mRNA^h*CFTR*^, for three different time points, normalized to mock-transfected cells. In pDNA^h*CFTR*^ transfected cells, the quenching efficacy was significantly higher after 48 h and stayed high even after 72 h (*P* ≤ 0.05), while mRNA^h*CFTR*^ as well as modified cmRNA^h*CFTR*^ transfected cells revealed a single peak quenching at 48 h (*P* ≤ 0.05), which was undetectable at 72 h in A549 cells. In CFBE41o- cells mRNA^h*CFTR*^ could not provide any detectable quenching but ${{\rm{cmRNA}}}_{{\rm{N}}1{{\rm{\Psi }}}_{1.0}/{\rm{m}}5{{\rm{C}}}_{1.0}}^{{\rm{h}}{CFTR}}$ produced significant quenching at all the time points (*P* ≤ 0.05) and ${{\rm{cmRNA}}}_{{\rm{s}}2{{\rm{U}}}_{0.25}/{{\rm{m5C}}}_{0.25}}^{{\rm{h}}{CFTR}}$ showed very significant quenching at 48 h (*P* ≤ 0.001), which is in line with expression patterns seen in Fig. 1A,B.

### (c)mRNA^h*CFTR*^ immunogenicity *ex vivo* by an adapted human whole blood assay

Due to lack of a reliable method to detect immune responses that therapeutic mRNAs may trigger in a living organism, we focused on an innovative approach to using whole blood from humans. Blood was collected from three healthy donors and used fresh to conduct whole blood assays. Interestingly, the negative control groups (blood only and NP only) did not raise IFN-α values above the detection limit (Fig. 2C, red dotted lines), while TNF-α and IL-8 were already measurable in human blood untreated or treated only with NPs. That is the reason why we adapted the graphical presentation, using a blue colored area that represents the variance of the negative controls, which are biological replicates. The positive control (R848) lead to a strong and significant production of IFN-α (6 h and 24 h, respectively; *P* ≤ 0.05), IL-8 (6 h and 24 h, respectively; *P* ≤ 0.01) and TNF-α (6 h and 24 h, respectively; *P* ≤ 0.05) (Fig. 2C). All cmRNA^h*CFTR*^ showed a very similar result in cytokine expression as observed for negative controls: the IFN-α levels did not reach the detection limit of the ELISA; IL-8 and TNF-α responses were not statistically significant at 6 h and 24 h, respectively (Fig. 2C). Unmodified mRNA^h*CFTR*^ resulted in a significant increase of IFN-α at 6 h and 24 (*P* ≤ 0.05), only significant increase in IL-8 at 24 hours (*P* ≤ 0.05) and the TNF-α levels were in line with the negative control. While pDNA^h*CFTR*^ triggered high TNF-α responses at 6 h (*P* ≤ 0.05), significant and detectable IFN-α and IL-8 responses after 6 h and 24 h (*P* ≤ 0.05). Due to both, significantly lower expression of mRNA^h*CFTR*^ *in vitro* (Fig. 1) and unwanted higher immune responses of mRNA^h*CFTR*^, we focused on cmRNA^h*CFTR*^ and pDNA^h*CFTR*^ in the following therapeutic studies.

### Therapeutic effect of cmRNA^h*CFTR*^*in vivo* in mice after i.t. and i.v. application

**Figure 3:**
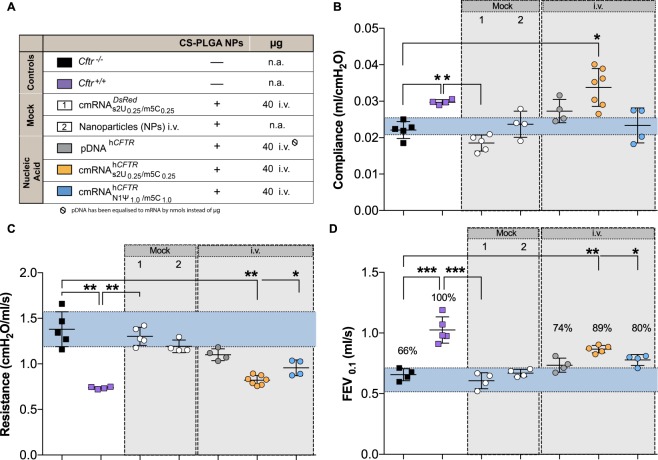
*In vivo* lung function measurements in cmRNA^h*CFTR*^ and pDNA^h*CFTR*^ treated *Cftr*^−/−^ mice by i.v. route. All mouse groups utilized in (**B–D**) are color-coded for their treatment schemes (**A**), including dosage and application routes. (**B–D**) Precision *in vivo* lung function measurements covering all relevant outcome parameters on in *Cftr*^−/−^ mice treated twice via i.v. route and measured 72 hours after the 2^nd^ instillment; *n* = 4–7 mice per group. The blue area represents the variance of the negative controls which are biological replicates. Data represent the means ± SD on compliance, resistance and Forced Expiratory Volume in 0.1 seconds (FEV_0.1_). *P ≤ 0.05; **P ≤ 0.01 and ***P ≤ 0.001 versus untreated *Cftr*^−/−^ mice.

**Figure 4:**
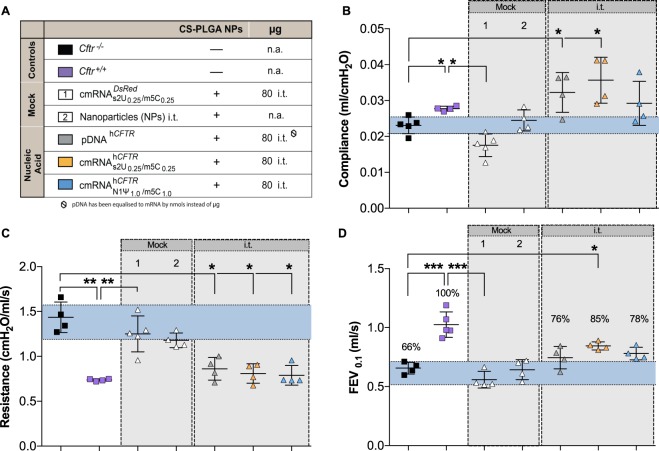
*In vivo* lung function measurements in cmRNA^h*CFTR*^ and pDNA^h*CFTR*^ treated *Cftr*^−/−^ mice by i.t. route. All mouse groups utilized in (**B–D**) are color-coded for their treatment schemes (**A**), including dosage and application routes. (**B–D**) Precision *in vivo* lung function measurements covering all relevant outcome parameters on *Cftr*^−/−^ mice treated twice via i.t route and measured 72 hours after the 2^nd^ instillment; *n* = 4–7 mice per group. The blue area represents the variance of the negative controls which are biological replicate. Data represent the means ± SD on compliance, resistance and Forced Expiratory Volume in 0.1 seconds (FEV_0.1_). *P ≤ 0.05; **P ≤ 0.01 and ***P ≤ 0.001 versus untreated *Cftr*^−/−^ mice.

All *in vivo* experiments were performed with nanoparticles if not stated otherwise. Therapeutic potential of cmRNA^h*CFTR*^ was investigated in a mouse model of Cystic Fibrosis. *Cftr*^−/−^ and *Cftr*^+/+^ mice have been used in several experimental settings that are explained and color-coded in Fig. 3A. To assess the impact of cmRNA^h*CFTR*^ on lung function, we evaluated clinically relevant parameters using the FlexiVent® lung function measurement system. We observed significant differences between Mock controls, *Cftr*^−/−^ and healthy wild-type mice for all parameters measured (*P* ≤ 0.05; Figs 3 and 4B, *P* ≤ 0.01; Figs 3 and 4C and *P* ≤ 0.001 Figs 3 and 4D). I.v. administration of ${{\rm{cmRNA}}}_{{\rm{s}}2{{\rm{U}}}_{0.25}/{{\rm{m5C}}}_{0.25}}^{{\rm{h}}{CFTR}}$ significantly increased the compliance from 0.02 ± 0.01 ml/cmH_2_O (*Cftr*^−/−^ mice) to 0.03 ± 0.01 ml/cmH_2_O (*P* ≤ 0.05), reaching equivalent values to those measured in *Cftr*^+/+^ mice (Fig. 3B). In contrast, the i.v. application of 40 µg ${{\rm{cmRNA}}}_{{\rm{N}}1{{\rm{\Psi }}}_{1.0}/{\rm{m}}5{{\rm{C}}}_{1.0}}^{{\rm{h}}{CFTR}}$ or pDNA^h*CFTR*^ did not alter compliance significantly. Applying 40 µg of ${{\rm{cmRNA}}}_{{\rm{s}}2{{\rm{U}}}_{0.25}/{{\rm{m5C}}}_{0.25}}^{{\rm{h}}{CFTR}}$ or ${{\rm{cmRNA}}}_{{\rm{N}}1{{\rm{\Psi }}}_{1.0}/{\rm{m}}5{{\rm{C}}}_{1.0}}^{{\rm{h}}{CFTR}}$ i.v. significantly lowered the resistance (*P* ≤ 0.01 and *P* ≤ 0.05 respectively, Fig. 3C) but pDNA^h*CFTR*^ did not alter the resistance at a significant level. FEV_0.1_ (human equivalent of FEV_1_) of *Cftr*^+/+^ mice defined as projecting 100% forced expiratory volume is pointedly different compared to the FEV_0.1_ value of *Cftr*^−/−^ mice of only 66% of the wild-type (*P* ≤ 0.001). I.v. injection of 40 µg ${{\rm{cmRNA}}}_{{\rm{s}}2{{\rm{U}}}_{0.25}/{{\rm{m5C}}}_{0.25}}^{{\rm{h}}{CFTR}}$ significantly improved the FEV_0.1_ by 23 percentage points (*P* ≤ 0.01) and i.v. injection of 40 µg ${{\rm{cmRNA}}}_{{\rm{N}}1{{\rm{\Psi }}}_{1.0}/{\rm{m}}5{{\rm{C}}}_{1.0}}^{{\rm{h}}{CFTR}}$ provided a significant FEV_0.1_ improvement of 14 percentage points compared to FEV_0.1_ value of untreated *Cftr*^−/−^ mice (*P* ≤ 0.05; Fig. 3D). However, i.v. administration of pDNA^h*CFTR*^ showed no significant improvement of FEV_0.1_. I.v. injected mock cmRNA^*DsRED*^ or nanoparticles alone encouragingly aligned with untreated groups in all determined lung function parameters. Taken together, these results demonstrate significant lung function improvement in all relevant lung function parameters of *Cftr*^−/−^ mice intravenously (i.v.) treated with cmRNA^h*CFTR*^.

In the i.t. treated groups, a substantial improvement in compliance and resistance could be detected when the ${{\rm{cmRNA}}}_{{\rm{s}}2{{\rm{U}}}_{0.25}/{{\rm{m5C}}}_{0.25}}^{{\rm{h}}{CFTR}}$ dose was increased to 80 µg (0.04 ± 0.01 ml/cmH_2_O and 0.86 ± 0.18 cmH_2_O.s/ml respectively; P ≤ 0.05; Fig. 4B,C). However, 80 µg of ${{\rm{cmRNA}}}_{{\rm{N}}1{{\rm{\Psi }}}_{1.0}/{\rm{m}}5{{\rm{C}}}_{1.0}}^{{\rm{h}}{CFTR}}$ i.t. lowered the resistance but did not improve the compliance as effectively as ${{\rm{cmRNA}}}_{{\rm{s}}2{{\rm{U}}}_{0.25}/{{\rm{m5C}}}_{0.25}}^{{\rm{h}}{CFTR}}$ (*P* ≤ 0.05; Fig. 4B,C). pDNA^h*CFTR*^ (80 µg) i.t. treated mice also produced significant improvements of resistance and compliance (P ≤ 0.05, Fig. 4B,C). In terms of FEV_0.1_, i.t. application of 80 µg ${{\rm{cmRNA}}}_{{\rm{s}}2{{\rm{U}}}_{0.25}/{{\rm{m5C}}}_{0.25}}^{{\rm{h}}{CFTR}}$ was improved by 19 percentage points and i.t. application of 80 µg ${{\rm{cmRNA}}}_{{\rm{N}}1{{\rm{\Psi }}}_{1.0}/{\rm{m}}5{{\rm{C}}}_{1.0}}^{{\rm{h}}{CFTR}}$ improved the FEV_0.1_ by 12 percentage points with respect to untreated *Cftr*^−/−^ mice (*P* ≤ 0.05, Fig. 4D). I.t. administration of pDNA^h*CFTR*^ showed no significant improvement of FEV_0.1_. Taken together, these results demonstrate significant lung function improvement in all relevant lung function parameters of *Cftr*^−/−^ mice treated intratracheally with cmRNA^h*CFTR*^.

**Figure 5:**
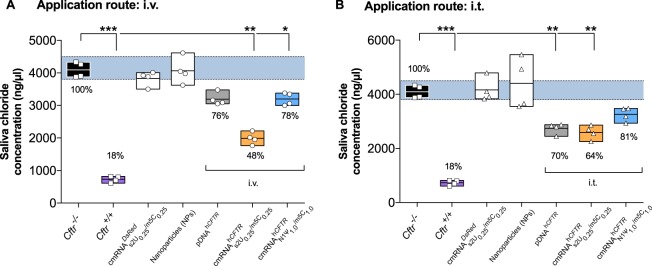
*In vivo* saliva chloride concentration measurement of cmRNA^h*CFTR*^ and pDNA^h*CFTR*^ treated *Cftr*^−/−^ mice by i.v./ i.t. route (**A**,**B**) Functional test of reconstituted CFTR channel and reduced chloride concentration after i.v. (**A**) or i.t. (**B**) treatment of *Cftr*^−/−^ mice compared to untreated *Cftr*^−/−^ (black), positive controls (violet), and percentages relative to the positive control; *n* = 4 mice per group; two mock controls were included (white); boxes represent the means ± minimum and maximum values. The blue area represents the variance of the negative controls which are biological replicates. *P ≤ 0.05; **P ≤ 0.01 versus untreated *Cftr*^−/−^ mice.

A well-established functional test, measuring the mouse saliva chloride concentration^33^ was conducted to complement the functional results observed using FlexiVent. The saliva chloride concentration detected in *Cftr*^−/−^ mice (4084 ± 236.8 ng/µl) was significantly higher compared to *Cftr*^+/+^ mice (748.8 ± 96.9 ng/µl, *P* ≤ 0.001; Fig. 5A,B). The treatment of ${{\rm{cmRNA}}}_{{\rm{s}}2{{\rm{U}}}_{0.25}/{{\rm{m5C}}}_{0.25}}^{{\rm{h}}{CFTR}}$ i.v. significantly lowered the chloride concentrations in the saliva of *Cftr*^−/−^ mice by more than 52 percentage points (*P* ≤ 0.01; Fig. 5A) underlining the FlexiVent results. However, ${{\rm{cmRNA}}}_{{\rm{N}}1{{\rm{\Psi }}}_{1.0}/{\rm{m}}5{{\rm{C}}}_{1.0}}^{{\rm{h}}{CFTR}}$ and pDNA^h*CFTR*^ treated mice (i.v.) only provided about 20 percentage points reduction. The treatment with ${{\rm{cmRNA}}}_{{\rm{s}}2{{\rm{U}}}_{0.25}/{{\rm{m5C}}}_{0.25}}^{{\rm{h}}{CFTR}}$ i.t. (80 µg) significantly lowered the chloride concentrations in the saliva of *Cftr*^−/−^ mice by 36 percentage points (*P* ≤ 0.01; Fig. 5B). ${{\rm{cmRNA}}}_{{\rm{N}}1{{\rm{\Psi }}}_{1.0}/{\rm{m}}5{{\rm{C}}}_{1.0}}^{{\rm{h}}{CFTR}}$ treated mice (i.t.) abridged the chloride concentration not significantly in saliva of *Cftr*^−/−^ mice but pDNA^h*CFTR*^ treated *Cftr*^−/−^ provided a significant reduction (*P* ≤ 0.01; Fig. 4B) but not as proficiently as ${{\rm{cmRNA}}}_{{\rm{s}}2{{\rm{U}}}_{0.25}/{{\rm{m5C}}}_{0.25}}^{{\rm{h}}{CFTR}}$. The mock mRNA treated group and just nanoparticle treated group failed to decrease the chloride concentration.

### cmRNA^h*CFTR*^ and hCFTR protein quantification in lungs after application *in vivo*

**Figure 6:**
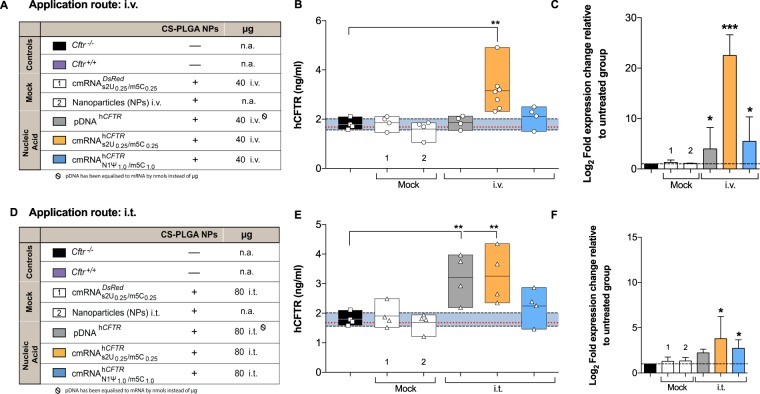
Expression of hCFTR protein in mouse lungs and delivery of cmRNA^h*CFTR*^ and pDNA^h*CFTR*^ in lungs. (**A**,**D**) All mouse groups, particles and particle combinations depicted in the study plan are color-coded for their treatment schemes, including dosage and application routes. (**B**,**E**) hCFTR ELISA, detecting specifically human CFTR, was performed on lung preparations at day 6 from *Cftr*^−/−^ mice treated twice via i.v. (**B**) or i.t. (**E**) route and measured 72 hours after the 2^nd^ instillment (endpoint); the same *n* = 4–7 mice per group were used. (**C**,**F**) Relative amounts of differently modified h*CFTR* mRNAs in the lungs applied i.v. or i.t., then determined by RT-quantitative PCR, compared to untreated *Cftr*^−/−^ mice (*P ≤ 0.05); *n* = 4–7 mice per group. All bar graph data are depicted as means ± SDs while box plots data are depicted as the means ± minimum to maximum values. The blue area represents the variance of the negative controls which are biological replicates. *P ≤ 0.05; **P ≤ 0.01 and ***P ≤ 0.001 versus untreated *Cftr*^−/−^ mice.

All *in vivo* experiments were performed with nanoparticles if not stated otherwise. We tested for the localization of cmRNA^h*CFTR*^ complexed with nanoparticle in the lungs after i.t. or i.v. application via RT-qPCR, quantified the hCFTR protein expression with hCFTR ELISA and then evaluated its immunogenicity depending on modification. In contrast to the *in vitro* data, when 40 µg ${{\rm{cmRNA}}}_{{\rm{s}}2{{\rm{U}}}_{0.25}/{{\rm{m5C}}}_{0.25}}^{{\rm{h}}{CFTR}}$ was i.v. injected into the mice, this resulted in a higher accumulation of that mRNA in the lung as compared to 40 µg ${{\rm{cmRNA}}}_{{\rm{N}}1{{\rm{\Psi }}}_{1.0}/{\rm{m}}5{{\rm{C}}}_{1.0}}^{{\rm{h}}{CFTR}}$ and pDNA^h*CFTR*^ (*P* ≤ 0.01, Fig. 6C). More importantly, we wanted to analyze if there is a significant increase in hCFTR protein levels in the lungs of treated mice by hCFTR ELISA (Fig. 6B,E). These analyses confirmed that mice treated with 40 µg ${{\rm{cmRNA}}}_{{\rm{s}}2{{\rm{U}}}_{0.25}/{{\rm{m5C}}}_{0.25}}^{{\rm{h}}{CFTR}}$ i.v. had a highly significant increase of hCFTR protein in the lungs of treated mice vs. control mice *(P* ≤ 0.01; Fig. 6B). Besides, we tested the effects of an increased amount (80 µg) of cmRNAs and pDNA^h*CFTR*^ with i.t. instillation, ${{\rm{cmRNA}}}_{{\rm{s}}2{{\rm{U}}}_{0.25}/{{\rm{m5C}}}_{0.25}}^{{\rm{h}}{CFTR}}$ and pDNA^h*CFTR*^ showed a clear and significant increase of hCFTR protein compared to control mice (Fig. 6E) (P ≤ 0.01). All the mock controls used in hCFTR ELISA have proven to be not significantly different from the negative control.

### cmRNA^h*CFTR*^ immunogenicity *in vivo* in mice after i.v. application

**Figure 7:**
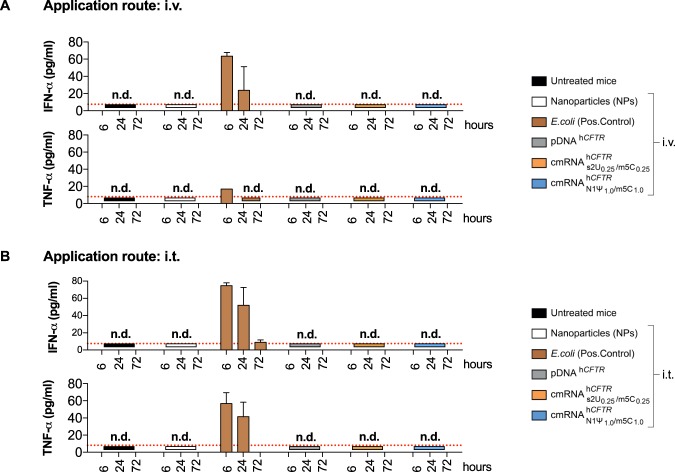
(c)mRNA^h*CFTR*^ and pDNA^h*CFTR*^ mediated immunogenicity *in vivo* Mice were i.v. or i.t. injected with a mix of (c)mRNA and NPs at a 1:10 ratio and ELISAs were performed post-i.v./i.t.-injection at three different time points. n.d., not detectable. The red dotted lines in (**A**,**B**) mark the detection limit as specified in the respective ELISA kit. All bar graph data are depicted as the means ± SD and box plots data are represented as the means ± minimum to maximum values.

All *in vivo* experiments were performed with nanoparticles if not stated otherwise. First, we applied different compounds such as nanoparticles, *E*. *coli* extract total RNA (positive control), cmRNA^h*CFTR*^ and pDNA^h*CFTR*^ i.v. or i.t. to mice and monitored their immune reaction at three different time points. Applying 40 µg cmRNA^h*CFTR*^ (with any modifications used) or pDNA^h*CFTR*^ i.v. or i.t. did not lead to detectable responses of key cytokines IFN-α or TNF-α (detected by ELISA) at all three-time points (Fig. 7A)^34,35^. Nanoparticles alone (used in all *in vivo* experiments) showed no immune response over the detection limit. However, as expected the positive control (*E*. *coli* extract total RNA) i.v. and i.t. resulted in a significant increase of IFN-α and TNF-α at 6 h and a trend increase of IFN-α at 24 h, while an effect at 72 h was not detectable (Fig. 7A). No immune response had been observed apart from positive control in groups treated intratracheally (i.t) (Fig. 7B).

## Discussion

Although much progress has been achieved since the discovery of the CFTR gene 25 years ago, there is still a substantial need to restore robust CFTR function in patients suffering from cystic fibrosis^8^. With the recent approvals of the small molecule agents ivacaftor and lumacaftor, science has paved a possible way to overcome the hurdles caused by the disease-conferring gene. Those treatments can be more or less effectively applied to patients bearing *CFTR* mutations delF508 (Lumacaftor-ivacaftor/Orkambi) and G551D (ivacaftor)^36–39^. However, lung function, as one of the main outcome parameters probably having the most significant influence on life quality of CF patients, is rarely tested in preclinical models. In fact, actual effects of (modern) existing drugs on lung function, with forced expiratory volume in one second (FEV_1_) as a key parameter, are quite low^40^. Here, by using cmRNA^h*CFTR*^, we are presenting a proof of concept for a viable and potent therapeutic alternative. We have vigorously tested mRNA therapy with focus on *in vivo* lung function normalization while avoiding any possible, unwanted immune responses for a possibility of repeated dosing. The unique formulation utilized can be used both topically (intratracheally) and systemically (via i.v. injection), having in both cases a profound effect on normalizing the lung function parameters, including compliance, resistance and FEV_0.1_ of treated *Cftr*^−/−^ mice to values obtained from *Cftr*^+/+^ mice.

*In vitro*, using cmRNA^h*CFTR*^, CFTR protein expression in CFBE41o− cells was increased up to 5.5-fold compared to mRNA^h*CFTR*^, which is consistent with previous studies obtained by us and others^18,31,41^. Incorporation of naturally occurring chemically modified nucleosides has been shown to suppress inhibitory effects on translation by avoiding detection by pattern recognition receptors (PRRs) such as Toll-like receptors (TLRs) TLR3, TLR7, and TLR8^34,35^. Those receptors play a crucial role in the detection, processing, and degradation of mRNA. Interestingly, depending on the mRNA modification, kinetics of hCFTR expression varies upon the different nucleosides used. In fact, after 72 h we only observe an increased quenching of Yellow fluorescent protein (YFP) in YFP assay in CFTR null A549 and CFBE41o− cells by pDNA^h*CFTR*^ which would corroborate our findings from our flow cytometry and western blot analyses in CFBE41o− cells. In contrast there is a significant increase in I^−^ influx by functional hCFTR channels and quenching of YFP at 48 h post transfection by cmRNA^h*CFTR*^. Consequently, we assume that upon different cell lines, kinetics by which the hCFTR protein is expressed varies. Earlier studies support our notion that differently modified mRNAs can have an impact on the translational effect between distinct cell lines^31,35^.

To better mimic *in vivo* human conditions, we performed an *ex vivo* whole blood assay (WBA) which offers a more complex environment to test for immune responses. This assay has already been used in a number of preclinical settings, and Coch and colleagues could demonstrate that it has the potential to reflect broad aspects of the *in vivo* cytokine release caused by oligonucleotides^42^. Indeed, we could show that the small molecule Resiquimod (serving as a positive control by activating TLR7 and TLR8) lead to a substantial release of IFN-α, TNF-α and IL-8. pDNA^h*CFTR*^, as well as unmodified mRNA^h*CFTR*^, also showed elevated cytokine levels probably due to the activation of innate immune receptors^34,35^. In contrast, incorporation of modified nucleosides into h*CFTR* mRNA (cmRNA^h*CFTR*^) abolished such responses, with no detectable amounts of IFN-α. This is in concert with previously published data, demonstrating cmRNA’s limited immune responses, mainly by evading detection from receptors such as TLRs, RIG-1, MDA-5 or PKR^34,41^. Interestingly, even though TNF-α or IL-8 could be detected, it rather shows donor-dependency than effects deriving from NPs and/or cmRNA^h*CFTR*^ with cytokine levels being all within the variance of negative controls. Although it mirrors only the blood compartment and does not reflect the more complex *in vivo* situation, the WBA can give a prediction of how cytokines are released in the human system in response to systemically applied (c)mRNA prior to clinical testing.

To determine the clinical potential of CFTR-encoded cmRNA we compared not only different modifications *in vivo* but also two different routes of administration. I.t. application has been chosen for this study on the base of our previous findings of applying cmRNA i.t. in a surfactant protein-B deficient mouse model leading to significantly prolonged survival^26^. Given the fact that in patients suffering from CF one of the key barriers is the airway mucus layer in which inhaled particles are more likely to get trapped and removed, we sought to apply cmRNA^h*CFTR*^/pDNA^h*CFTR*^ complexed to NPs by i.v. injection as an alternative administration route. Systemic delivery via lipid-modified polymeric nanoparticles have been already shown to target the lungs efficiently^43^.

To support our notion of improved CFTR activity, we performed extensive lung function measurements using state-of-the-art technology to provide detailed *in vivo* information on different lung function parameters. There are doubts about *Cftr*^−/−^ mice as a proper model for cystic fibrosis as it does not reflect the typical lung phenotype seen in CF patients^44^. However, the reason behind that seems to be in how deeply lungs or other affected organs had been investigated. A layer of material can be observed with characteristics of an acid mucopolysaccharide on the bronchiolar surface and is also evident in alveoli by using scanning electron microscopy in *Cftr*^−/−^ mice, which is not evident in *Cftr*^+/+^ mice^45^. It has also been reported *Cftr*^−/−^ mice shows similar effect of CF patients like, age-dependent pulmonary inflammation, death of respiratory epithelial cells and high vulnerability to severe *Pseudomonas aeruginosa* infection^46^. Recent studies could demonstrate reduced airway compliance and increased resistance in comparison to wild-type mice^47,48^. Indeed, we observed significantly higher and lower levels regarding resistance and compliance, respectively, in *Cftr*^−/−^ controls and mock-treated *Cftr*^−/−^ mice compared to homozygous wild-type mice (*Cftr*^+/+^) mice and demonstrated that treatment with cmRNA^h*CFTR*^-NPs improved compliance and resistance significantly equal to those seen in healthy *Cftr*^+/+^ mice. FEV_1_ percentage (for mouse or small animal FEV_0.1_) is related to survival in CF and a most important physiological parameter for CF patients. A previous study demonstrated that patients with a %FEV_1_ of <30 compared to healthy individuals had a 50% chance of mortality within 2 years and hence are regularly examined in clinical setup^49^. A strong variance amid *Cftr*^−/−^ controls and mock-treated *Cftr*^−/−^ mice compared to homozygous wild-type mice (*Cftr*^+/+^) mice has been perceived in the case of FEV_0.1_. Our study provides a significant improvement of FEV_0.1_ due to treatment with NP-cmRNA^h*CFTR*^. Interestingly, NP-pDNA^h*CFTR*^ when administered via i.t. route improved parameters of lung function measurements including FEV_0.1_, but not as significant as cmRNA^h*CFTR*^. We also observed i.v. or i.t. administration of ${{\rm{cmRNA}}}_{{\rm{s}}2{{\rm{U}}}_{0.25}/{{\rm{m5C}}}_{0.25}}^{{\rm{h}}{CFTR}}$ to positively compensate most of lung function parameters. Overall, we could demonstrate that certain protocols, applying cmRNA^h*CFTR*^ either i.v. or i.t. efficiently restored lung function values equal to those of wild-type. Suggesting a more even distribution through arteries and the bronchial circulation by i.v. injection, this route and formulation could lead to a very potent therapy especially for newborns and young infants. By providing functional CFTR early in life, the lungs could be protected from irreversible damage. Nevertheless, when applied intratracheally, which mimics deep inhalation of a spray or powder formulation (primary application route in adults), an adjustment in dose and/or formulation (e.g. ${{\rm{cmRNA}}}_{{\rm{s}}2{{\rm{U}}}_{0.25}/{{\rm{m5C}}}_{0.25}}^{{\rm{h}}{CFTR}}$ increased to 80 µg) might easily abrogate any negative effect of the *Cftr*^−/−^ genetic background on lung function.

Eventually, we determined the impact of cmRNA^h*CFTR*^ and pDNA^h*CFTR*^ on another relevant physiological outcome such as the saliva chloride concentration to evaluate therapeutic effect and complement the lung function results. Sweat chloride concentration has become an accepted method as a diagnostic readout to assess treatment effects of CF patients^50^. As an analog, chloride concentration of β-adrenergic stimulated salivary glands of *Cftr*^−/−^ mice can be investigated as it complies with findings in CF patients^33^. In this study, we could show a substantial difference in salivary Cl^−^ content of cmRNA^h*CFTR*^ and pDNA^h*CFTR*^ treated mice – both, i.v. and i.t. – compared to their untreated counterpart. With end point-analysis, a significant decrease in Cl^−^ to nearly 50% was observed, indicating a restoration of CFTR in the duct compartment of salivary glands and thus leading to an improved Cl^−^ absorption. Previous studies estimated that a restoration of CFTR activity to 50% could lead to sweat chloride levels to near normal levels in CF patients. Given that, it is possible that cmRNA^h*CFTR*^ treatment has the potential to improve CFTR activity to levels that are at least similar to those in patients with a mild CF phenotype^51^.

In this study, by applying cmRNA^h*CFTR*^ consecutively, both modifications were successfully delivered to the lungs with the i.v. route being more efficient at doses of 40 µg (2 mg/kg body weight) per treatment. Intriguingly, in contrast to the results obtained *in vitro*, ${{\rm{cmRNA}}}_{{\rm{s}}2{{\rm{U}}}_{0.25}/{{\rm{m5C}}}_{0.25}}^{{\rm{h}}{CFTR}}$ showed a significantly higher CFTR protein expression with higher accumulation of h*CFTR* mRNA in lung cells. Assuming differences of cmRNA-encoded transgene expression between distinct cell lines, it is plausible to consider such differences between *in vitro* versus *in vivo* applications, which is by far more complex. In this respect, the higher amount of ${{\rm{cmRNA}}}_{{\rm{s}}2{{\rm{U}}}_{0.25}/{{\rm{m5C}}}_{0.25}}^{{\rm{h}}{CFTR}}$ found in lung cells after i.v. injection, might be due to the fact that its nucleoside composition is more favorable to evade PRRs, thus being less degraded. However, regardless of cmRNA kinetics we also observed differences in the delivery route of cmRNA^h*CFTR*^/pDNA^h*CFTR*^ -NPs. Our data suggest i.v. injection to be more efficient in delivering such complexes to the lung than topical administration. Tests of cmRNA^h*CFTR*^ -NP’s capacity of mucus penetration are in planning phase including detection of cmRNA^h*CFTR*^ and CFTR protein (glycosylated) in a *Cftr*-deficient mouse model especially at the apical side of the bronchial epithelium. The upper airways are lined with mucus and mucociliary movements clear foreign particles immediately. In addition, the main barriers in the deeper areas are the alveolar lining, scavenger transporters and alveolar macrophages^52,53^. We, therefore, concluded that the original dosing by which cmRNA-NPs were delivered i.t. was not as efficient as using the i.v. route. Indeed, increasing the amount by doubling the dose (to 80 µg) for each treatment showed a hCFTR expression close to levels seen using the i.v. route.

To exclude immune reactions caused by either NPs or the cmRNA^h*CFTR*^ itself, we conducted extensive immune assay tests *in vivo*. Except for the positive control (*E*. *coli* total mRNA), we could not detect any immunostimulatory effect *in vivo* that could arise from NPs or the cmRNA^h*CFTR*^. These results confirm our previous studies in which we showed that NPs, as well as modified mRNA, could be administered safely to the lungs without any substantial increase in cytokines, or inflammatory-related cells such as macrophages or neutrophils^26^. Systemic delivery has also been reported to have no impact on proinflammatory cytokine secretion^29^.

Taken together, this study is the first proof of concept of efficient application of NP-cmRNA^h*CFTR*^ *in vivo* to restore lung function in a *Cftr*-deficient mouse model. Importantly, we could neither detect immune responses *in vivo* nor in a more defined setting *ex vivo*. Applying cmRNA^h*CFTR*^ to *Cftr*^−/−^ mice could efficiently restore lung function close to levels of healthy control mice. In addition, our study compared - apart from two well-known mRNA modifications and pDNA^h*CFTR*^ - also two different delivery routes, demonstrating that systemic administration of cmRNA targets lung cells more efficiently at lower dosages. This study provides a proof of concept for alternative treatment of patients suffering from CF. cmRNA^h*CFTR*^ transcript supplementation may be broadly applicable for most *CFTR* mutations, not only in adults but already in the postnatal state, thereby protecting the lungs from exacerbations from the very beginning of life.

## Electronic supplementary material

Supplementary data
