# Supplementary material for: Chemically modified hCFTR mRNAs recuperate lung function in a mouse model of cystic fibrosis
Source: Sci Rep. 2018 Nov 13;8:16776. doi: 10.1038/s41598-018-34960-0 (PMC6233194; doi:10.1038/s41598-018-34960-0)
Supplement: Supplementary file 1 — Supplementary data [file 41598_2018_34960_MOESM1_ESM.pdf]

Chemically modified h*CFTR* mRNAs recuperate lung function in a mouse model of cystic fibrosis

Authors: AKM Ashiqul Haque[1], Alexander Dewerth[1], Justin S. Antony[1,7], Joachim Riethmüller[2†], Georg R. Schweizer[1], Petra Weinmann[1], Ngadhnjim Latifi[1], Hanzey Yasar[3], Nicoletta Pedemonte[4], Elvira Sondo[4], Brian Weidensee[1], Anjali Ralhan[5], Julie Laval[5], Patrick Schlegel[1], Christian Seitz[1], Brigitta Loretz[3], Claus-Michael Lehr[3,6], Rupert Handgretinger[1,7], Michael S. D. Kormann[1*]

Supplement figures:

Figure-S1

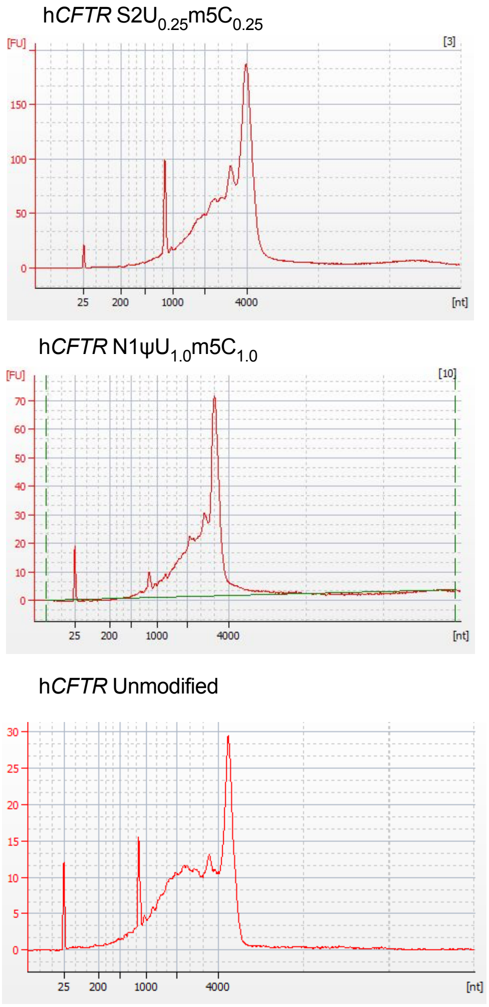

Figure-S1: Bioanalyzer data after *in vitro* transcription (IVT) of hCFTR (c)mRNA shows the size of (c)mRNA^{hCFTR} (Around 4Kb).

Figure-S2

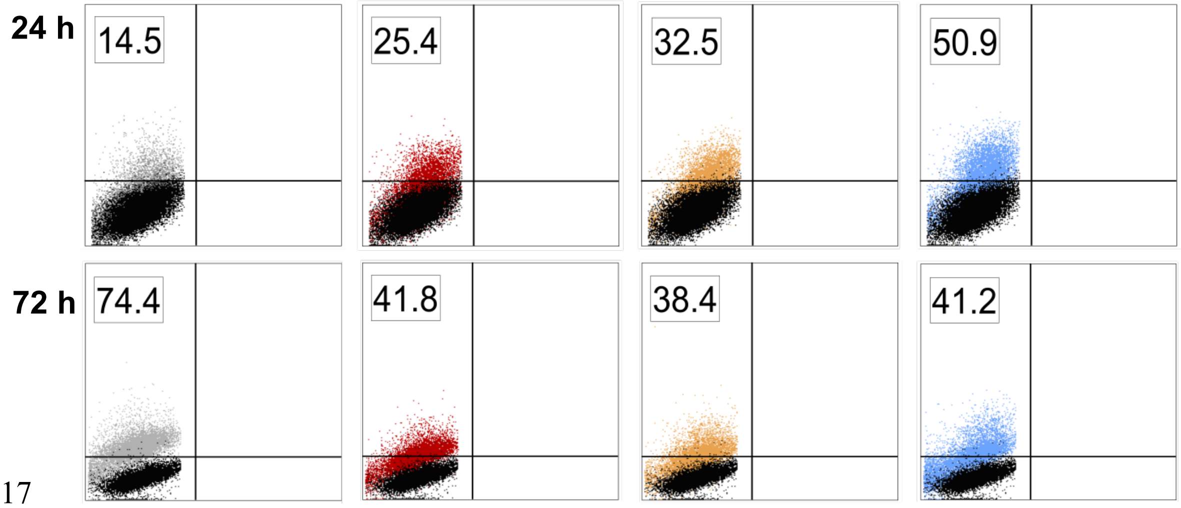

24h	Treatment	Sample #	% pos cells	MFI	Total expression
	pDNA^{hCFTR}	1	14,5	15,0	217,5
		2	15,6	15,5	241,8
		3	17,2	16,6	285,5
	mRNA^{hCFTR}	1	25,4	22,8	579,1
		2	21,0	17,3	363,3
		3	24,6	19,9	489,5
	cmRNA$^{hCFTR}_{s2U_{0.25}/m5C_{0.25}}$	1	32,1	32,4	1040,0
		2	33,0	33,0	1089,0
		3	35,7	35,3	1260,2
	cmRNA$^{hCFTR}_{N1\Psi_{1.0}/m5C_{1.0}}$	1	50,9	57,2	2911,5
		2	48,0	55,1	2644,8
		3	50,0	56,1	2805,0
**72h**	Treatment	Sample #	% pos cells	MFI	Total expression
	pDNA^{hCFTR}	1	80,6	14,2	1140,5
		2	72,9	13,8	1007,5
		3	75,3	13,4	1007,5
	mRNA^{hCFTR}	1	52,7	9,3	488,0
		2	55,8	10,0	555,2
		3	54,2	9,4	507,3
	cmRNA$^{hCFTR}_{s2U_{0.25}/m5C_{0.25}}$	1	45,8	7,8	355,9
		2	42,6	8,0	372,3
		3	47,1	8,8	416,4
	cmRNA$^{hCFTR}_{N1\Psi_{1.0}/m5C_{1.0}}$	1	48,6	7,9	385,4
		2	45,3	8,8	434,3
		3	44,8	6,4	305,6

Figure-S2: Expression analyses of hCFTR protein by flow cytometry; n=3

Figure S3

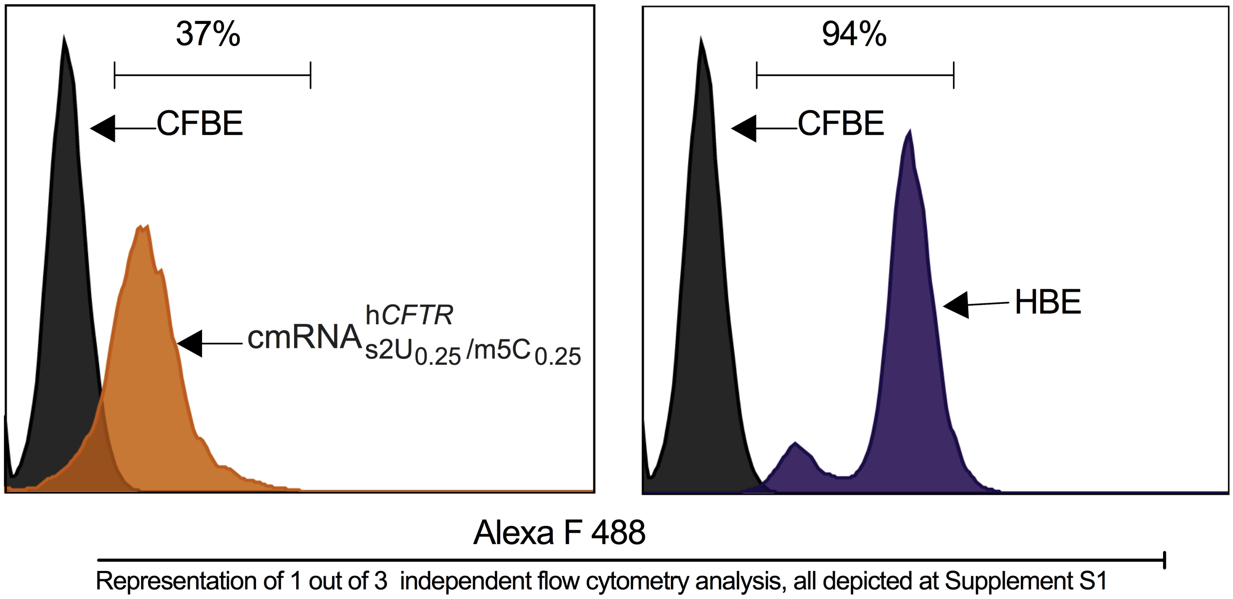

Figure-S3: Expression analyses of hCFTR protein between CFBE41o–, transfected CFBE41o– and 16HBE14o- by flow cytometry.

Figure S4:

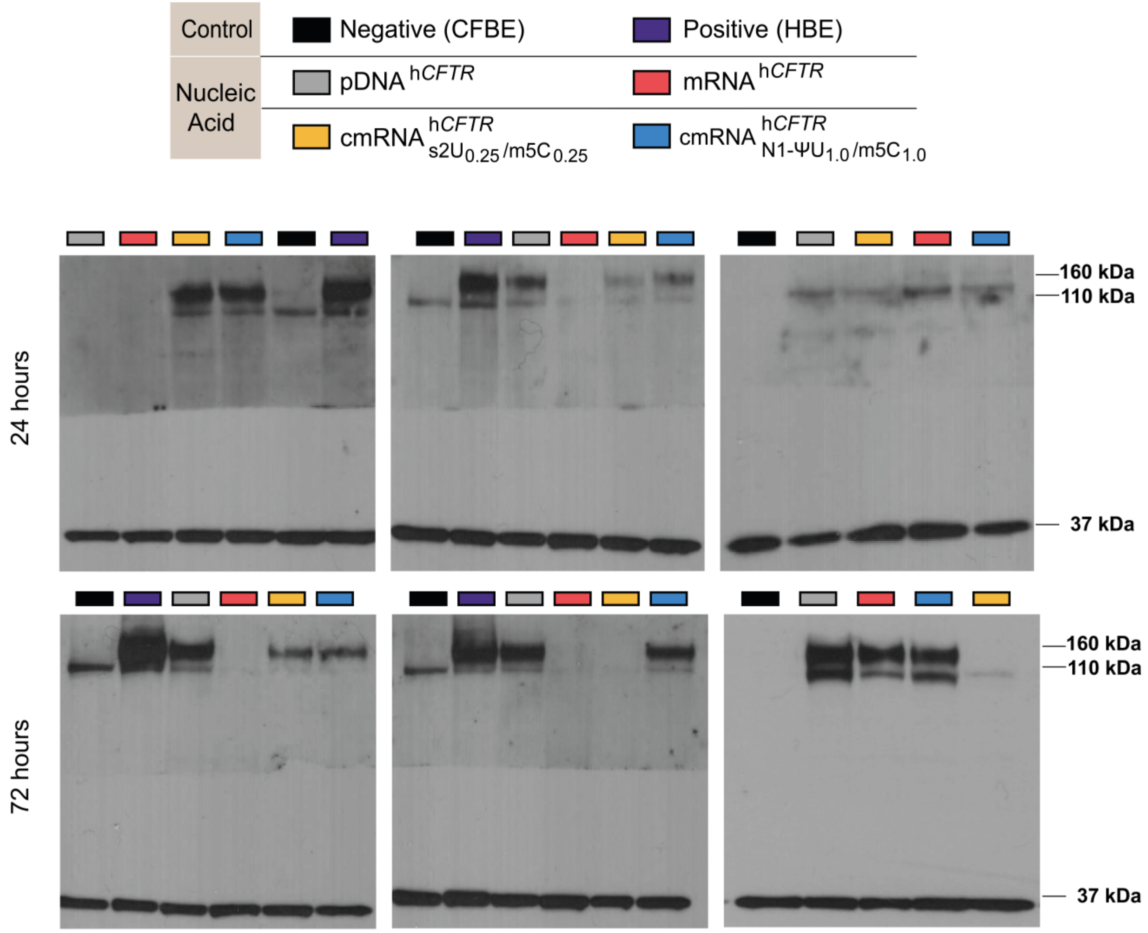

Figure. S4: Western blot analyses of hCFTR protein in CFBE41o- and 16HBE14o- cells; n=3. All the membranes have been exposed for 30 mins. The Membranes were cut at 80 kDa so GAPDH and hCFTR can be stained and detected simultaneously without using membrane stripping technique. Before developing the membrane for imaging both parts of the membrane were reassembled.

Table T1

Chitosan-PLGA NPs	*Size (nm)*	*PDI*	*Zeta potential (mV)*
batch 1	137,5	0,11	19,4
batch 2	144,9	0,131	21,4
batch 3	143,3	0,131	24,8
Mean	141,9	0,124	21,9
SD	3,9	0,012	2,7

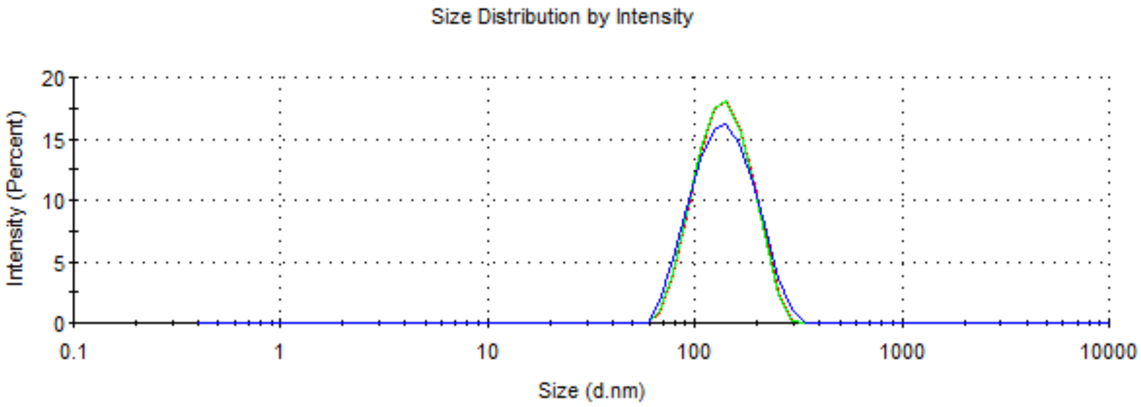

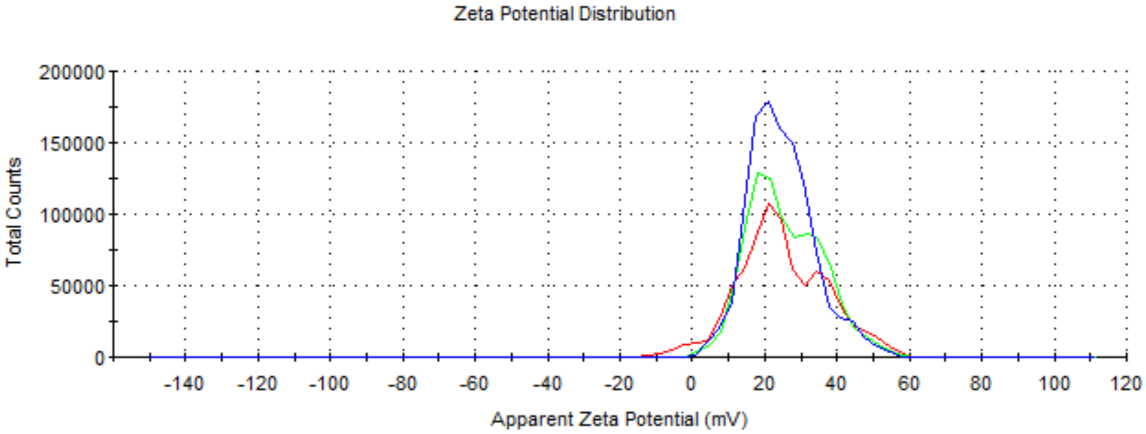

Table T1: Physiochemical characterization of Chitosan-PLGA nanoparticle

Figure S5

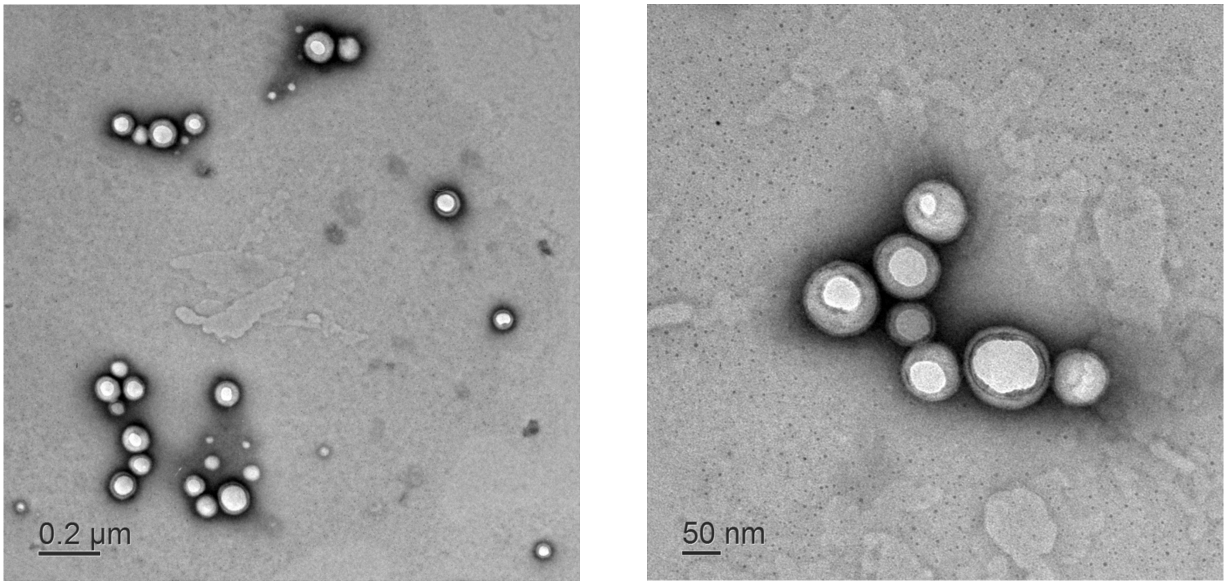

Figure S5: CS-PLGA nanoparticles under electron Microscope stained with 0.5% phosphotungstic acid solution.
